# Human Laboratory Models of Cannabis Use: Applications for Clinical and Translational Psychiatry Research

**DOI:** 10.3389/fpsyt.2021.626150

**Published:** 2021-02-25

**Authors:** Reilly R. Kayser, Margaret Haney, Helen Blair Simpson

**Affiliations:** ^1^Department of Psychiatry, Columbia University Vagelos College of Physicians and Surgeons, New York, NY, United States; ^2^Research Foundation for Mental Hygiene, New York State Psychiatric Institute, New York, NY, United States

**Keywords:** cannabis (marijuana), cannabinoids, psychiatric disorders, anxiety disorders, human laboratory research, clinical and translational research

## Abstract

Cannabis is increasingly used by individuals with mental health diagnoses and often purported to treat anxiety and various other psychiatric symptoms. Yet support for using cannabis as a psychiatric treatment is currently limited by a lack of evidence from rigorous placebo-controlled studies. While regulatory hurdles and other barriers make clinical trials of cannabis challenging to conduct, addiction researchers have decades of experience studying cannabis use in human laboratory models. These include methods to control cannabis administration, to delineate clinical and mechanistic aspects of cannabis use, and to evaluate potential treatment applications for cannabis and its constituents. In this paper, we review these human laboratory procedures and describe how each can be applied to study cannabis use in patients with psychiatric disorders. Because anxiety disorders are among the most common psychiatric illnesses affecting American adults, and anxiety relief is also the most commonly-reported reason for medicinal cannabis use, we focus particularly on applying human laboratory models to study cannabis effects in individuals with anxiety and related disorders. Finally, we discuss how these methods can be integrated to study cannabis effects in other psychiatric conditions and guide future research in this area.

## Introduction

Societal attitudes and public policy regarding cannabis use have shifted dramatically over the past two decades. Americans increasingly view cannabis as harmless (1), and as of December 2020, 36 states and the District of Columbia (DC) have legalized medicinal cannabis, while 15 states and DC permit recreational cannabis use. As acceptance of cannabis grows, more Americans are using, with 4.8 million more adults reporting near-daily cannabis use in 2018 compared to 2008 (2). Meanwhile, cannabis is purported to treat a variety of ailments. Despite limited evidence to support many of these claims, cannabis products are increasingly marketed as treatments for various medical conditions, including psychiatric disorders (3, 4). Thus, there is an urgent need to examine cannabis' purported mental health benefits and rigorously test its effects in patients with psychiatric disorders. In this paper, we describe how investigators can apply human laboratory methods to address these issues.

### Why Study the Effects of Cannabis in Psychiatric Populations?

Americans increasingly use cannabis medicinally (i.e., with the intent to treat one or more symptoms) (5). Psychiatric symptoms including anxiety, depression, and stress are among the most common reasons for which patients report seeking medicinal cannabis (6, 7). Of 9,003 American adults who responded to a randomized, nationally-representative survey, 7% endorsed medicinal cannabis use, among whom 47 and 39%, respectively used cannabis to treat anxiety and depression (5). Among 2,774 American cannabis users in another survey, 13.6 and 12.7% reported using cannabis as a substitute for anxiolytics and antidepressant medication, respectively (8). Cannabis use to treat psychiatric symptoms may have further increased during the COVID-19 pandemic: Among 1,202 American medicinal cannabis users surveyed before and after the pandemic's onset, self-reported cannabis consumption increased by 91% more for those with mental health conditions vs. those without (9).

Although interest in, access to, and use of cannabis is increasing, surprisingly few studies have directly examined its effects in those with medical or psychiatric illness. As a result, there is limited evidence supporting using cannabis as a treatment. A 2017 report by the National Academies of Sciences, Engineering, and Medicine found sufficient evidence to support treating only two conditions with medicinal cannabinoids (i.e., cannabis constituents or analogs): Chronic pain (for which both oral cannabinoids and cannabis showed efficacy) and multiple sclerosis-related spasticity (for which only oral cannabinoids were effective) (10). The psychiatric literature is even sparser: A 2019 meta-analysis including 88 studies found insufficient evidence to support medicinal cannabis treatment of any psychiatric condition (7).

Despite this dearth of research, individuals with psychiatric illness increasingly use cannabis for both recreational and medicinal purposes (11). Yet lacking evidence of cannabis' effects on psychiatric symptoms, patients and clinicians have little information to guide decisions about how to use it. Whereas, FDA approval for medications requires release of detailed information about different doses, routes of administration, indications, and potential adverse effects, this information for a highly variable plant is largely unknown. Advertising from the now-billion-dollar cannabis industry has filled the information void and often includes claims about cannabis' purported psychiatric benefits (3, 12). Meanwhile, cannabis is a federally-illegal substance legalized by individual states, with scant consensus regarding how it should be used. For instance, a physician may recommend medicinal cannabis to a patient with PTSD in New York, but not in Iowa, while physicians in Colorado have discretion to recommend cannabis for any condition they determine it might help (including psychiatric illnesses) (13, 14). This creates a landscape that is bewildering to clinicians, who generally report feeling ill-equipped to make evidence-based recommendations about cannabis (15), and to patients, who may develop unrealistic expectations about what cannabis can and cannot do.

### Regulatory and Scientific Challenges to Studying Cannabis Use in Human Volunteers

Clinical trials of cannabis are challenging to conduct in the US, partially due to cannabis' Schedule I labeling by the Drug Enforcement Agency (DEA). Researchers studying cannabis and other Schedule 1 substances must obtain special federal and state licensure (which can take months to years) following extensive monitoring and DEA-approved storage procedures (e.g., storing cannabis in a gun safe) (14). Thus, cannabis studies are typically limited to highly-specialized research environments. Moreover, current researchers may only use cannabis produced by the National Institute of Drug Abuse (NIDA) (14); in contrast, a range of cannabis products are sold in dispensaries and other commercial outlets or can be obtained illicitly (and may differ substantially from NIDA cannabis) (16).

In addition to regulatory factors, several scientific issues complicate studies of cannabis in human volunteers. Because cannabis is most often smoked or vaporized, different patterns of inhalation can lead to substantial variability in serum cannabinoid concentrations compared to, for example, intravenous administration (17, 18). Standardized methods to administer cannabis can minimize this variability. Smoking and vaporizing involve different preparation and delivery procedures than oral or intravenous administration (e.g., inserting plant material into a cigarette), requiring researchers to use different blinding techniques with these methods.

Cannabis' effects are susceptible to expectancy, such that individuals who anticipate receiving active cannabis but instead receive placebo nonetheless report experiencing cannabis-like effects (19). Psychiatric symptoms are also responsive to placebos, even those administered without deception (20). Thus, placebo control is essential for cannabis trials in psychiatric populations. Because many participants may be familiar with cannabis effects (for example, 16% of all Americans were estimated to have used cannabis in the past year in 2018) (2), placebo selection is also important to consider.

Dissecting the mechanistic properties and clinical effects of cannabis can also be difficult. Cannabis is pharmacologically diverse, containing over 140 unique chemical constituents (“phytocannabinoids”). Many phytocannabinoids are likely psychoactive, and the neurobiological mechanisms of even the two best-studied, Δ-9 tetrahydrocannabinol (THC) and cannabidiol (CBD), are incompletely understood (21). The properties of different cannabis varietals vary with their phytocannabinoid composition, the form, dose, and frequency in which they are administered, and the users' history of cannabinoid exposure (22). Disentangling the contributions of these factors can be difficult outside of controlled settings.

While few of cannabis' potential clinical benefits have been rigorously tested, its abuse potential has been well-documented (23). This poses an additional challenge to its study in individuals with psychiatric illnesses [who may be at increased risk for developing cannabis use disorder (CUD), among other adverse effects] (24). Investigators need to consider designs that can distinguish between cannabis' effects on psychiatric symptoms (e.g., anxiolysis/anxiogenesis) and unrelated drug effects (e.g., intoxication), while also minimizing the risk that participants develop CUD or experience other cannabis-related harms.

Given the barriers involved in clinical research, cannabis' effects on psychiatric outcomes have mostly been examined through observational studies and surveys (7, 25, 26). These studies tend to rely on participants' retrospective self-reports of cannabis effects, which are subject to recall biases; in recruiting medicinal cannabis users (who by definition believe cannabis to be potentially helpful), they also involve selection bias. As noted above, both cannabis effects (19) and psychiatric symptoms (20) are influenced by expectancy. Given its pharmacologic diversity (22), accounting for the different effects of cannabis' various constituents (e.g., THC vs. CBD) is daunting even in controlled studies. In observational research, it is nearly impossible: Labeling of commercially-available cannabis products is frequently inaccurate (27, 28), state-run cannabis testing facilities have demonstrated systematic differences in the cannabinoid concentrations they report, and even experienced cannabis users have difficulty determining the THC/CBD content of the products they use from their subjective responses (29, 30). Further, cannabis that is smoked or vaporized vs. taken orally in tinctures or capsules will produce markedly different plasma cannabinoid concentrations (31).

Though observational research and surveys can be useful tools, their limitations make them insufficient to fully elucidate cannabis' clinical risks and benefits or its potential role in psychiatric treatment. Randomized, placebo-controlled trials remain the gold-standard tests of efficacy, yet only a few have examined cannabis' potential medicinal properties (of which only a subset involved patients with psychiatric disorders). Although small trials have tested psychiatric applications of synthetic cannabinoids (32) [e.g., nabilone, a synthetic THC analog that is approved by the US Food and Drug Administration (FDA) for treating cancer chemotherapy and HIV-related nausea and vomiting] and cannabinoid isolates (33) (e.g., various CBD preparations), recreational and medicinal users overwhelmingly ingest cannabinoids through inhaling smoked or vaporized cannabis flower (6, 16). While understanding cannabis' effects when used as it is most commonly in daily settings is critically important, a 2016 systematic review identified only one cannabis trial for any psychiatric indication (34). This open-label trial of smoked cannabis for PTSD lacked a placebo control or systematic method of cannabis administration (35). Since then, we have conducted two small placebo-controlled studies of smoked cannabis at our site: One tested its effects in individuals at high risk for psychotic disorders (36), and another tested its effects in patients with obsessive-compulsive disorder (OCD) (37).

### Rationale for Using Human Laboratory Methods to Study Cannabis Use in Psychiatric Populations

Given the current political, social, medical, and legal climate, the public health need for controlled studies of cannabis effects in psychiatrically ill populations has never been more urgent. Whereas, psychiatric cannabis trials are nascent, addiction researchers have explored cannabis effects in human laboratory studies for decades (38). Human laboratory methods were developed to study problematic use of psychoactive drugs like cannabis and to identify new ways of treating individuals with substance use disorders. These procedures enable investigators to study and control methods of administration and to blind participants/investigators for rigorous testing of clinical effects. Researchers have also devised strategies to delineate factors contributing to the development and maintenance of CUD and other substance use disorders. Finally, the human laboratory has proved to be an efficient venue in which to screen for potential therapeutic effects of psychoactive substances like cannabis and cannabinoids before testing them in large-scale clinical trials. Herein, we review some of these human laboratory methods and describe how they could be applied to examine the effects of cannabis and cannabinoids in patients with psychiatric illnesses.

## Using Human Laboratory Methods to Study the Effects of Cannabis and Cannabinoids in Psychiatric Populations

**Overview**: Substance use researchers have developed human laboratory methods to directly examine the effects of cannabis and its constituents. These include methods to control cannabis administration (e.g., dosing and blinding procedures), to delineate clinical and mechanistic aspects of cannabis use (e.g., intoxication and other acute effects, positive and negative reinforcement, dose-dependency, and tolerance), and to evaluate potential treatments (e.g., screening potential uses of cannabis in psychiatric treatment, testing treatments for comorbid psychiatric illness and CUD, and identifying cannabis-drug interactions). Below, we review these human laboratory procedures and describe their potential applications to explore cannabis effects in patients with psychiatric illnesses. Because anxiety disorders are among the most common psychiatric illnesses affecting American adults (39), and anxiety relief is also the most commonly-reported reason for medicinal cannabis use (5), we focus particularly on how human laboratory procedures could be applied to study cannabis effects in individuals with anxiety and related disorders. These procedures and associated applications are summarized in Table 1.

**Table 1:** Human laboratory procedures to model cannabis use and potential applications in patients with anxiety disorders.

**Category**	**Model**	**Advantages**	**Challenges**	**Publications**	**Example applications in patients with anxiety disorders**
Cannabis administration	Dosing	• Cued-dosing may improve standardization of cannabis delivery, while *ad-libitum* administration may reduce anxiogenic effects • Both procedures generate clinically-relevant effects • Methods exist to administer smoked, vaporized, and edible cannabis	• Cued-smoking may not reflect cannabis use in daily settings, while *ad-libitum* administration may increase variability in serum cannabinoid levels • Currently only NIDA-produced cannabis is permitted in human subjects research	Ramesh et al. (40) Haney et al. (41) Bidwell et al. (42)	*-Ad libitum* administration may generate sufficient cannabis exposure while mitigating potential anxiogenic effects from cued-dosing
	Blinding	• Reduce ability of investigators and participants to determine drug condition assignment • Limit observation of differences between laboratory-administered and naturalistically-used cannabis	• Participants may still detect psychoactive properties of cannabis • Difficult to design active controls	Chait et al. (43, 44) Kirk et al. (45) Metrik et al. (19)	-Careful attention to participant selection (e.g., excluding heavy cannabis users) and instructions (e.g., notifying participants that they will smoke cannabis containing a range of phytocannabinoids contents with varied effects on anxiety) may limit blinding failure
Clinical and mechanistic aspects of cannabis use	Intoxication & acute effects	• Measuring acute response to cannabis administration can help to establish a timecourse for cannabis effects • Can incorporate subjective (e.g., self-report) and objective (e.g., computerized cognitive tasks) measures to track outcomes of interest	• Effects may vary based on prior exposure to cannabis • Response may differ with long-term or repeated administration 3. Few current options for measuring rapid changes in psychiatric symptoms	Hart et al. (46) Vadhan et al. (47) Ramaekers et al. (73)	- Incorporating a visual analog scale to probe for rapid changes in anxiety symptoms
	Positive reinforcement & reward	1. Self-administration paradigms can model cannabis use to increase positive affect 2. Can examine reinforcement differences based on cannabinoid content and relative to other reward outcomes (e.g., food, money)	1. May be difficult to disentangle increased positive affect vs. decreased negative affect	Haney et al. (48) Hart et al. (49) Cooper and Haney (50)	- Comparing cannabis self-administration among anxious and non-anxious participants - Comparing self-administration of cannabis vs. benzodiazepines in anxious participants
	Negative reinforcement & withdrawal	1. Withdrawal/abstinence paradigms can model cannabis use to mitigate negative affect • Abstinence paradigms may be less logistically/ethically challenging to conduct than cannabis administration paradigms • Can also incorporate tasks to measure intoxication effects on negative affect	• Often requires inpatient admission 2. Differentiating negative affect related to withdrawal vs. psychopathology may be difficult	Metrik et al. (51) Hefner et al. (52, 53) Haney et al. (54)	- Assessing cannabis effects on tasks indexing anxiety states (e.g., the NPU task, which indexes startle response to predictable vs. unpredictable threat) in cannabis users with anxiety disorders. - Could assess participants' response to cannabis intoxication or compare effects of continued use vs. abstinence
	Dose-dependence & Tolerance	• Repeated-administration designs can identify tolerance to physiological and intoxication effects • Can also examine effects of different doses/concentrations	• Some acute cannabis effects (e.g., on impulse control, etc.) may persist even with repeated administration – tolerance to psychotropic effects is not clearly established	Haney et al. (55) Hart et al. (46) Ramaekers et al. (73)	- After first showing acute cannabis effects on psychiatrically-relevant outcomes, studies can explore whether the magnitude of these effects declines with continued administration - Exploring for dose-dependency by comparing cannabis varietals with varied concentration of THC and other phytocannabinoids
Potential pharmacological treatments	Therapeutic applications of cannabis	• Can address the critical need for placebo-controlled studies of cannabis effects • Within-subjects, repeated-measures designs allow trials to be conducted efficiently with adequate statistical power even at modest sample sizes • Human laboratory as a translational bridge to screen promising therapeutic applications for cannabis prior to investing in large-scale clinical trials • Allow testing of cannabis' behavioral, physiological, psychological, and neurocognitive targets, which is aligned with current NIMH initiatives to identify objective measures of psychopathology and enhance clinical trials	• Controlled laboratory environments may not reflect cannabis use in naturalistic settings • Researchers are currently restricted to using NIDA-produced cannabis • Participants in laboratory studies may differ from general psychiatric populations 4. Different challenges depending on illness being studied (e.g., risk for psychosis in individuals with bipolar disorder)	Vadhan et al. (36) Kayser et al. (37)	- Assessing acute effects of cannabis in individuals with anxiety disorders by first administering a single dose of cannabis followed by repeated assessments of self-reported anxiety, cardiovascular measures (e.g., heart rate), and threat response - Using within-subjects designs to compare the effects of different cannabis varietals (e.g., high-THC, high-CBD) vs. placebo
	Treatments for comorbid CUD + psychiatric illness	• Treatments for shared symptoms of CUD and psychiatric disorders can be evaluated in models of relapse or abstinence from cannabis use • Examining discrete outcomes related to cannabis use (e.g., withdrawal, relapse) and psychopathology (e.g., symptom self-report) can clarify the mechanistic basis for treatment effects	• Disentangling effects of CUD vs. psychopathology may be challenging (e.g., self-reported anxiety due to GAD vs. cannabis withdrawal)	Haney et al. (41, 56–59) Herrmann et al. (60)	- Assessing medications to treat symptoms of GAD and cannabis withdrawal (e.g., anxiety, irritability, restlessness, insomnia) in cannabis users with GAD - To clarify mechanism for any observed medication effects, outcomes to be examined might include. Cannabis self-administration, anxiety self-report, and threat response.
	Drug-drug interactions with cannabis and/or cannabinoids	• Cannabis contains >140 phytocannabinoids and thus could interact with many commonly-used medications • Laboratory procedures can screen for such interactions under controlled conditions • Can also determine whether preclinical evidence for cannabis-drug interactions replicates in human subjects	• Potential drug-drug interactions for the vast majority of phytocannabinoids are largely unexplored even in preclinical studies	Hartman et al. (61) Gaston et al. (62) Alsherbiny and Li (63)	- Assessing serum levels of anxiolytic medications (e.g., SSRIs) following coadministration with cannabis (or cannabinoids)

### Methods to Control Cannabis Administration

#### Procedures to Control Dosing

**Figure 1:**
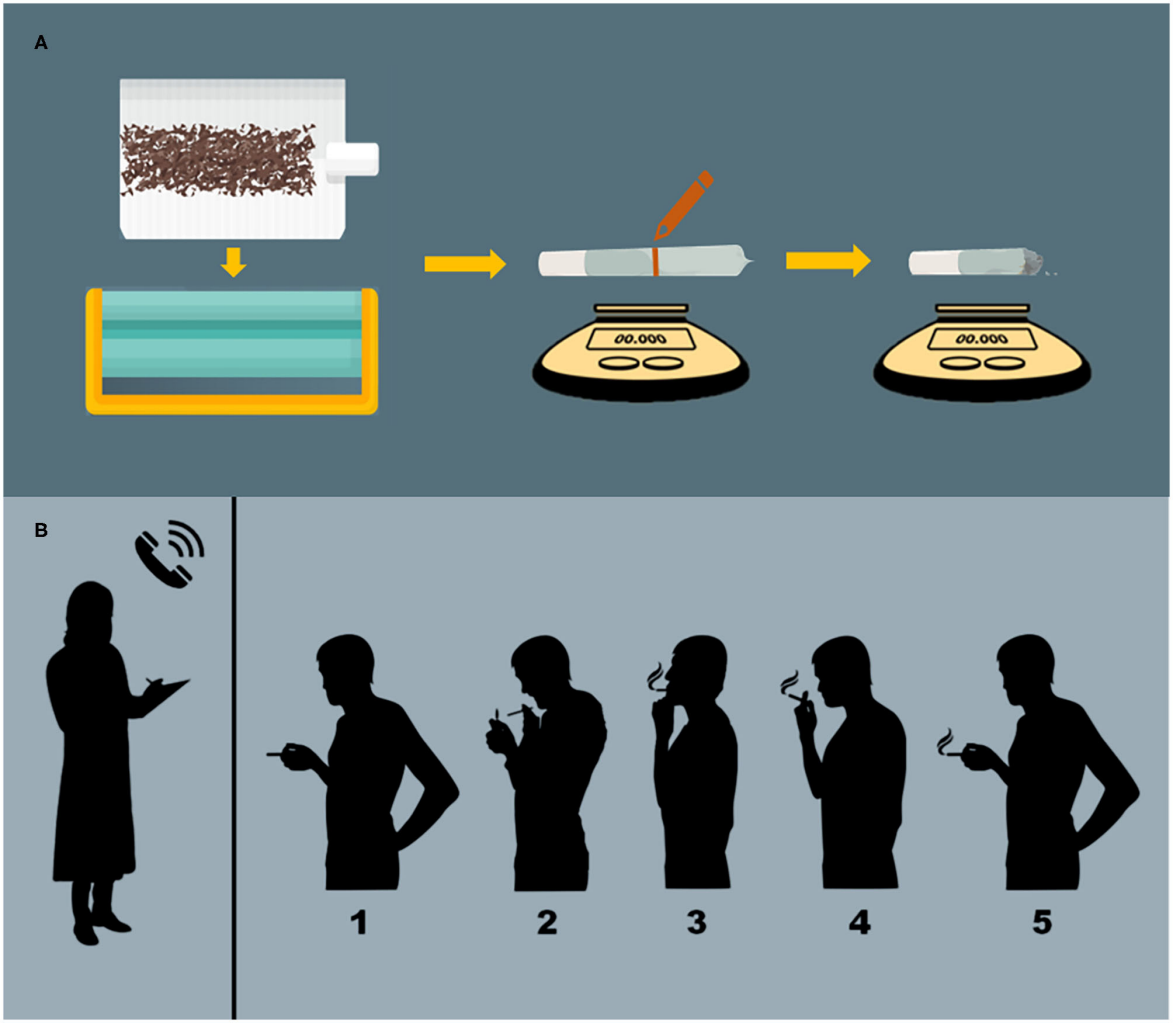
Administration procedures. **(A)** Preparation of cannabis cigarettes. Cannabis cigarettes are machine-rolled using cigarette paper and then inserted into a plastic cigarette holder. A line is drawn at the half-way point and the participant is instructed to smoke 50% of the cigarette. Cannabis consumption is verified via pre- and post-administration weighing of cigarettes. **(B)** Cued-smoking procedure. From a separate room with a two-way mirror, an investigator (who has no other contact with participants) guides participants through cued-smoking procedures. (1) The participant is presented with the cannabis cigarette, and then instructed to (2) “Prepare” (light the cigarette and prepare to smoke), (3) “Inhale” (5 s), (4) “Hold smoke in lungs” (10 s), and (5) “Exhale.” This cycle is repeated, allowing a 40 s interval between puffs, until 50% of the cigarette is pyrolized.

Cued-smoking procedures have been developed to help standardize cannabis administration (64). Investigators provide participants a specific amount of cannabis containing known concentrations of constituents (e.g., THC, CBD), and then guide them through the process of smoking, controlling the duration of inhalation and the amount of time that smoke is held within the lungs (see Figure 1 for details). Similar methods exist for controlled administration of vaporized (31, 65) and edible (31) cannabis formulations. Following cannabis administration, participants' subjective, physiological, and/or neurocognitive responses can be measured at precise timepoints (38).

Though potentially improving standardization, cued-smoking procedures may not reflect how cannabis is used in daily settings. Moreover, asking participants to smoke a specific percentage of a cannabis cigarette (rather than allowing them to titrate to their desired level) may induce discomfort or anxiety in some individuals if, for example, they are required to smoke more cannabis than they are comfortable smoking (51). Some studies have accounted for such effects by instructing participants to smoke *ad libitum* over the course of a predefined time period (e.g., 10 min) (42, 66). A*d libitum* cannabis administration may increase variability in serum cannabinoid concentrations, but recent studies suggest it nonetheless yields clinically-relevant effects (42, 66–68). Thus, in a study of patients with anxiety disorders, *ad libitum* procedures may generate sufficient cannabis exposure while also mitigating potential anxiogenic effects due to administration procedures (rather than cannabis itself) that might occur with cued-smoking.

Despite attempts to standardize administration procedures, cannabis smokers adjust their inhalation patterns as a function of cannabinoid content (i.e., decrease inhalation as THC content increases, and vice versa) (40, 69). As a result, both cued-smoking and *ad libitum* administration yield relatively consistent serum cannabinoid concentrations, even when accounting for differences in potency (i.e., THC content) (69). Nonetheless, participants experience clinically-relevant effects when guided through these smoking procedures. Indeed, even heavy users who are tolerant to cannabis will become intoxicated from controlled administration of low-potency cannabis in the human laboratory (41).

#### Procedures to Improve Blinding

Placebo-controlled trials assume that participants and investigators are blinded to drug conditions (i.e., that inactive and active agents are indistinguishable). Blinding is critical in cannabis research because cannabis users experience significant expectancy effects when exposed to cannabis-related cues (e.g., cigarette appearance and smell, the act of smoking) (43, 45, 70), and also report subjective cannabis-like effects when they anticipate receiving active cannabis but instead receive placebo (19). Moreover, participants' observation of differences between laboratory-administered cannabis and the cannabis they use outside of the lab may influence expectancy (71). As described above, psychiatric symptoms are also particularly sensitive to expectancy effects; thus, adequate blinding is essential to studying cannabis effects in psychiatric illness. Fortunately, human laboratory researchers have developed extensive procedures to improve blinding to cannabis dosing conditions (44).

In the cannabis administration procedures outlined above, blinding is maintained through the following methods (detailed in Figure 1): (36, 37, 41). First, cigarettes are machine-rolled using cigarette paper. They are then inserted into a plastic cigarette holder and a line is drawn at the half-way point, after which the cigarette is presented to the participant. The participant is then guided through the smoking procedure until 50% of the cigarette is smoked (verified by pyrolization to the half-way mark on the cigarette). Smoking only half of a cigarette prevents participants and investigators from seeing the color of its contents (which might vary across conditions or differ from the cannabis participants use in daily settings) and masks the moisture content of the cigarette (which affects burn time and may be higher in placebo vs. active cannabis). Smoking through a plastic cigarette holder also prevents participants from squeezing and possibly occluding the end of the cigarette with their lips, and ensures more consistent puff-to-puff delivery of smoke components, which vary (often increase) with successive puffs (44). Once participants have smoked to the 50% mark, consumption can also be verified via pre- and post-administration weighing of cigarettes (41).

Another approach to the blinding problem is to instruct participants that they will smoke cannabis containing a wide range of THC and other cannabinoids, some of which are intoxicating and others which are not, and ask them to guess their treatment assignment after study completion (72). Across a variety of human laboratory studies (19, 69), individuals receiving placebo cannabis often guess that they instead received a low-potency (but still active) varietal, suggesting the presence of expectancy effects. Investigators can also assess participants' self-report of psychological and physiological effects from active vs. placebo cannabis (19, 40). Other proposed approaches have included recruiting cannabis-naïve participants, which may improve blinding but also potentially increase risk for addiction and other adverse effects (e.g., panic attacks), or using active controls, which may be challenging in that it is unclear which substance suitably mimics the effects of cannabis (euphoria, dry mouth, tachycardia, etc.) without affecting other relevant outcomes (71). Finally, using within-subjects designs, investigators can compare different cannabis varietals with varied concentrations of THC and other cannabinoids (36, 37) while also reducing participants' ability to determine their assigned condition by increasing the range of phytocannabinoids concentrations they could possibly receive.

The blinding approaches above could easily be applied to study how cannabis affects individuals with anxiety disorders. That said, the instructions participants receive should be designed carefully to limit potential expectancy effects on self-reported anxiety: For example, investigators may inform patients that they will smoke cannabis with different concentrations of THC/CBD (rather than active cannabis vs. placebo), which may have a range of effects on anxiety (rather than being anxiolytic or anxiogenic). Excluding heavy cannabis users (e.g., weekly or greater) may reduce the chances that experienced participants guess their assigned condition (in addition to mitigating tolerance effects); to limit risk for adverse cannabis effects, researchers could recruit participants with at least some prior experience using cannabis without negative effects (e.g., >1 lifetime use without experiencing a panic attack).

### Methods to Dissect Clinical and Mechanistic Aspects of Cannabis Use

#### Intoxication and Other Acute Effects

Acute cannabis effects can be examined in laboratory studies by obtaining self-reports, physiological assessments, and/or neurocognitive tests at specific intervals following cannabis administration; these methods also permit exploration of cannabis' acute effects on psychiatric outcomes. Cannabis studies typically ask participants to self-report ratings of intoxication, including how “high” they feel, cannabis “liking,” and “good/bad effect.” Because THC produces dose-dependent increases in heart rate, researchers often integrate serial physiological assessments to establish a timeline for acute cannabis effects. Laboratory studies have also included repeated self-report assessments to probe acute changes in psychiatric symptoms: (36, 37). For example, patients with OCD in our cannabis trial were asked to complete standardized scales of obsessions, compulsions, and anxiety following cannabis administration (37). Other studies have used computerized cognitive tasks [administered once (46) or serially (47)] or obtained neuroimaging assessments (73) to examine acute cannabis effects on neurocognitive outcomes.

Selecting appropriate self-report instruments may be challenging for psychiatry researchers, since many validated scales measure symptoms over long-term (i.e., weeks to months) rather than rapid timeframes (i.e., minutes to hours) (74). While better ways to assess acute changes in psychiatric symptoms are needed, pending their development, studies of rapid-acting treatments (e.g., ketamine) often use a simple visual analog scale (VAS) to identify symptomatic changes (75, 76). In the above laboratory study in patients with OCD, we used a VAS to explore patients' self-report of change in obsessions and compulsions (on a scale from 1 to 10); (37) similar measures could easily be developed to explore cannabis-related symptomatic changes in patients with anxiety or other psychiatric disorders.

#### Positive and Negative Reinforcement

Behavioral pharmacology studies in non-treatment seeking cannabis smokers demonstrate that cannabis is positively reinforcing: Given the option to self-administer different cannabis varietals in a laboratory setting, participants will administer THC-containing cannabis more often than cannabis containing minimal THC (50). Depending on THC content, participants in these paradigms will also choose to receive THC-containing cannabis over non-drug alternatives like money (49) or a preferred food (48). The *incentive-sensitization* model describes how positive reinforcement may contribute to increased cannabis use among those with psychiatric illness: Individuals who associate cannabis with pleasure develop greater motivational salience toward cannabis-related cues, which elicits more approach behaviors and attentional bias toward cannabis cues that ultimately increase the likelihood of further cannabis use (77). Several psychiatric conditions including attention-deficit-hyperactivity disorder (ADHD) involve deficits in motivation and attention, reflecting dysfunction in reward-related (particularly dopaminergic) neural circuits (78, 79). Individuals with such deficits may be more susceptible to positive reinforcement from cannabis, which is consistent with epidemiological data supporting higher rates of cannabis use for those with untreated ADHD than in the general population (80).

To date, most laboratory investigations of cannabis' capacity for positive reinforcement have been in cannabis users or adults with CUD. However, self-administration paradigms could also be used to delineate cannabis-related positive reinforcement effects in participants with psychiatric disorders. One example would be for researchers to compare self-administration of cannabis among adults with anxiety disorders and controls matched for their patterns of cannabis use. Another would be to offer anxious participants the choice to receive either cannabis or anxiolytic medications known to be positively-reinforcing (e.g., benzodiazepines) (81).

There is also substantial evidence that cannabis is negatively reinforcing, meaning that individuals use it to escape or reduce the effects of aversive states (e.g., negative affect, withdrawal) (82). Laboratory models of cannabis-associated negative reinforcement typically focus on withdrawal states, admitting participants to an inpatient unit where their access to cannabis is controlled and/or stopped completely (54, 83) and then assessing symptoms of cannabis withdrawal (e.g., disrupted sleep, negative mood) and self-administration. These procedures also have identified differences in cognitive (e.g., reward valuation) (52) and physiological processes (threat response) (53) between cannabis users and controls. Specifically, compared to non-users, heavy cannabis users who abstained from cannabis for 3 days showed greater uncertainty aversion on a reward valuation task (52), while both abstinent and non-abstinent cannabis users had increased startle responses to unpredictable threat (a physiological marker of anxiety states) (53).

According to the *affect-motivational model*, negative reinforcement drives cannabis use by some individuals with affective psychopathology (e.g., depression/anxiety disorders), who may use cannabis situationally to attenuate affective symptoms (82). Supporting this idea, both depressive and anxiety disorders are linked to higher-than-average rates of cannabis use (82), and alleviating depression/anxiety symptoms is among the most commonly-cited reasons for which individuals seek medicinal cannabis treatment (5, 84). Moreover, preliminary neuroimaging data in both cannabis users (85) and non-cannabis using healthy volunteers (86, 87) suggest that THC acutely reduces functional activity in brain regions involved in emotional processing, particularly when evaluating negative face emotions.

Laboratory probes for negative reinforcement could test whether cannabis use alleviates symptoms or other aversive states in individuals with specific psychiatric diagnoses. Investigators might do this by assessing for differences in disease-relevant outcomes (e.g., symptom self-report, physiological measures, neurocognitive task performance) under conditions of continued use vs. abstinence, or following active vs. placebo cannabis administration. In the case of anxiety disorders, the neutral/predictable/unpredictable shock (NPU) task offers an example of an outcome that is sensitive to both disease- and cannabis-related effects. The NPU task, which indexes startle response to unpredictable vs. predictable threat, can discriminate between anxiety and fear states (88), has been used to screen for the effects of anxiolytic medications (89), and has identified effects related to cannabis withdrawal along with differences between cannabis users and controls (53). The task could easily integrate into laboratory models of intoxication or withdrawal, providing a powerful tool to evaluate for cannabis-related effects on anxiety.

#### Dose-Dependency and Tolerance

Dose-dependent cannabis effects have also been identified using human laboratory procedures (40, 90). These studies consistently find that cardiovascular outcomes and (to a lesser extent) self-rated subjective responses are sensitive to variation in THC content (40). Dose-response relationships for subjective responses have been more difficult to establish, possibly due to stronger influence of expectancy effects on self-report outcomes. Performance on error-monitoring tasks (e.g., the Flanker task) and other neurocognitive measures has also been shown to vary with THC dose (90).

Tolerance to the effects of THC-containing cannabis develops rapidly over the course of a few days. Cannabis users who were admitted to an inpatient unit where they received smoked cannabis initially reported acute increases in euphoria and intoxication (e.g., “high,” “good drug effect”), but the magnitude of these effects declined over several days of repeated administration. Moreover, tolerance developed dose-dependently (i.e., was greater when high-THC cannabis was administered compared to low-THC) (55). Tolerance to cannabis' physiological effects (e.g., tachycardia) developed dose-dependently over a similar timeframe in other studies (46). In contrast, cannabis' effects on neurocognitive functions like impulse control may persist even with sustained administration (91).

Similar designs could help to determine whether dose-dependency or tolerance moderate cannabis effects on psychiatrically-relevant outcomes. One strategy would be to recruit individuals with anxiety disorders to receive several cannabis varietals with varied THC content to determine whether THC dose moderates self-reported anxiety, blood pressure/heart rate, and or cognitive/physiological measures (e.g., the NPU task). If dose-dependent THC effects are identified, investigators could then assess whether tolerance develops following repeated administration. Establishing whether dose-dependency or tolerance occur will be critical in determining cannabis' potential role in treating anxiety or other psychiatric symptoms.

### Methods to Evaluate Pharmacological Treatments

Laboratory procedures already used to screen for CUD treatments can also be applied to study cannabis' role in psychiatric treatment, specifically by screening for potential uses of cannabis to treat symptoms of psychiatric disorders, evaluating medications to treat comorbid psychiatric and CUD symptoms, and assessing for cannabis-drug interactions. Examples of each application are provided below.

#### Potential Uses of Cannabis to Treat Psychiatric Illness

The human laboratory can serve as a translational bridge to move promising preclinical findings into clinical studies of cannabis. In this regard, a critical use for laboratory paradigms is to test the safety, tolerability, and clinical effects of cannabis in psychiatrically ill individuals. Findings from observational cannabis studies are often difficult to apply in real-world clinical scenarios because (as described above) these designs rarely capture the types of cannabis participants use, or how they ingest it. In contrast, human laboratory procedures permit delivery of precise amounts of cannabis in various forms (e.g., smoked, vaporized, or edible) and containing known phytocannabinoid concentrations. As a result, investigators are able to more accurately determine how the dose, formulation, and contents of cannabis relate to its clinical effects.

Human laboratory paradigms can also be used to validate cannabinoids' hypothesized targets. For example, investigators might test how cannabis acutely modulates brain function using task-based fMRI, or alters cognitive or physiological outcomes during paradigms like the NPU task. Evidence that cannabis meaningfully changes these outcomes could inform mechanistic understanding of its effects in psychiatric illness and may suggest potential treatment applications for further testing.

Finally, the human laboratory is an ideal venue for conducting preliminary tests of cannabis' efficacy as a psychiatric treatment. By incorporating placebo control and rigorous blinding procedures, laboratory paradigms are better able to count for expectancy effects than observational studies or surveys. Compared to clinical trials, human laboratory studies are also faster, cost less, and enable tighter control over potential confounds. Many use within-subjects designs that can achieve adequate statistical power with a smaller number of participants, facilitating testing of clinical effects, mechanistic hypotheses, and potential response moderators [e.g., age (92), gender (67, 93), genetics (94, 95), psychiatric history (96), and prior cannabis exposure (97)]. Laboratory models can thus function as a key intermediary step between preclinical research and clinical trials, rapidly generating data about the odds that cannabis treatment will succeed, which would then guide decisions about the utility of large-scale, resource-intensive clinical trials (98).

#### Treatments for Comorbid Psychiatric Disorders and CUD

Psychiatric comorbidity is common among adults with CUD, and conversely, psychiatrically-ill individuals are at greater-than-average risk for CUD (24). Though few laboratory studies have explored treatments for these combined conditions, CUD-relevant outcomes have been modeled extensively in the laboratory. These include relapse, operationally defined in the human laboratory as self-administration of cannabis following a period of abstinence. Though it would be unethical to offer cannabis to individuals seeking treatment for cannabis use, relapse can be modeled in non-treatment seeking cannabis users. Participants are typically admitted to an inpatient unit where they remain abstinent for several days. Then, they are given the choice to purchase individual puffs of a cannabis cigarette. Money not spent on cannabis self-administration is given to participants at study end. The initial puff, which reflects “relapse” to cannabis use, carries the greatest cost, while the cost of subsequent puffs decreases (56). Around 50% of participants in studies following these procedures will choose to “relapse”; (38) thus, investigators can explore how treatments influence the decision to resume cannabis use (56–59). Using similar methods, future studies might explore whether medications (e.g., SSRIs) moderate risk for cannabis relapse among individuals with anxiety disorders and CUD.

Cannabis self-administration models have also been used to test medications targeting symptoms of cannabis withdrawal. In one such paradigm, daily cannabis users, abstinent from cannabis for several days, were treated with nabilone at either 6 mg or 8 mg/day vs. placebo. Nabilone improved withdrawal-associated irritability and insomnia while significantly reducing the choice to pay money to self-administer cannabis following abstinence (i.e., a laboratory model of relapse) (56). A follow-up study found that adding zolpidem to nabilone more robustly targeted insomnia, with this combination yielding improved negative mood, anorexia, and insomnia while decreasing cannabis relapse rates compared to placebo or zolpidem alone (60). Both cannabis withdrawal and generalized anxiety disorder (GAD) involve symptoms of anxiety, irritability, restlessness, and insomnia, which may lead those with GAD to experience withdrawal symptoms more frequently or intensely, increasing their risk for continued cannabis use and relapse (99). Thus, using similar laboratory methods, investigators could examine medications or psychotherapies hypothesized to effectively treat these shared symptoms.

Finally, laboratory researchers have evaluated potential treatments to help individuals with CUD achieve abstinence. A straightforward approach used in many studies is to provide non-treatment-seeking cannabis users with either a medication or placebo, and then assess for between-group differences in cannabis self-administration (i.e., whether cannabis use is maintained, reduced, or stopped) (41, 56). Researchers have also used this procedure to explore the abstinence-promoting effects of contingency management paradigms (which offer participants monetary incentives to abstain from cannabis) (100) and cognitive behavioral therapy (CBT) (101). One preliminary study found that a modified form of CBT targeting both anxiety and CUD symptoms reduced self-reported anxiety and cannabis use among individuals with CUD and anxiety disorders; (102) future studies might examine whether this intervention moderates laboratory models of abstinence in this population.

#### Drug-Drug Interactions Between Cannabis and Psychotropic Medications

Two substances administered simultaneously may interact by pharmacodynamic (i.e., affecting the same receptor or target) and/or pharmacokinetic (i.e., affecting absorption, distribution, metabolism, or excretion) mechanisms. The most commonly reported drug-drug interactions involve pharmacokinetic changes to the activity of cytochrome P450 (CYP450) enzymes, leading to altered drug metabolism. With over 140 phytocannabinoid constitutents (103), cannabis can potentially interact with a range of medications. Animal studies suggest that THC and CBD be substrates for and inducers/inhibitors of CYP450 enzymes (63). With a diverse array of targets including 5HT_1A_ receptors, CBD also has a variety of potential pharmacodynamic interactions with psychotropic drugs (104).

While not all drug-drug interactions identified in animal models are clinically relevant, human trials of both THC and CBD have shown that they interact with common medications. In patients with epilepsy, co-administration of CBD modified serum levels of various antileptics including topiramate, clobazam, and zonisamide (62). Conversely, in adult cannabis users, alcohol increased serum THC levels when co-administered with cannabis (61). Preliminary studies also suggest that cannabis and its constituents can interact with warfarin, oxymorphone, disulfiram, pentobarbital, and cocaine, among other agents (63). Interactions between cannabis/cannabinoids and most psychotropic medications (including anxiolytics) have not been rigorously tested. The human laboratory may be an ideal venue to assess for these potential interactions under controlled conditions.

### Integrating Human Laboratory Procedures to Study Cannabis Effects in Psychiatric Illness: Example From a Study in Adults With OCD

Our human laboratory study of smoked cannabis in adults with OCD offers one example of how these paradigms could be applied to screen for therapeutic cannabis effects and inform future clinical and translational research (37). Considering preclinical evidence that cannabinoids affect key cognitive processes and neural circuits implicated in OCD (105), along with anecdotal reports from our clinic patients who suggested that cannabis relieved their symptoms, we conducted a randomized, placebo-controlled, within-subjects study. Twelve adult participants with OCD received three cannabis varietals over the course of three laboratory sessions: High-THC (7.0% THC/0.18% CBD), high-CBD (0.4% THC/10.4% CBD), and placebo (0% THC/CBD). Cannabis was administered using cued-smoking procedures, and serial measurements of OCD symptoms, state anxiety, intoxication, and cardiovascular measures were obtained over 3 h. We found that OCD symptoms and state anxiety decreased immediately following cannabis administration in all three conditions. However, there were no differences in OCD symptoms as a function of cannabis condition. Further, placebo cannabis yielded greater reductions in state anxiety than either active varietal. High-THC cannabis significantly increased heart rate and self-reported intoxication compared to both high-CBD and placebo, demonstrating that the cannabis exposure was sufficient to produce physiological and subjective effects.

This human laboratory study integrated several of the procedures reviewed above, including cued-smoking and blinding methods, self-report scales measuring psychiatric symptoms and intoxication, and physiological assessments. Findings have important clinical, public health, and research implications. Our data suggest that smoked cannabis may have little short-term benefit to individuals with OCD, which would argue against clinical use of cannabis as an acute OCD treatment, inclusion of OCD among the indications for physician-recommended cannabis, or conduct of large-scale clinical trials of smoked cannabis for the acute treatment OCD. Alternatively, finding acute benefits from active cannabis over placebo would have supported further study of its potential clinical utility in OCD: This might have included laboratory examinations of the potential risks and benefits of longer-term cannabis use in OCD (i.e., repeat administration over days to weeks), larger-scale trials assessing its acute efficacy for treating OCD symptoms, or mechanistic studies exploring the basis for the preliminary clinical effects that were observed. Because our preliminary study did not support these larger trials, we were able to quickly move on to pursue alternative research directions.

We then asked a different empirical question: Can THC facilitation of extinction improve the efficacy of existing therapeutic approaches? In a small pilot trial, we tested the effects of 4 weeks of daily treatment with nabilone (an FDA-approved synthetic THC analog) in patients with OCD, and found that nabilone had little effect on OCD symptoms as monotherapy, but appeared to enhance the effects of exposure-based psychotherapy when both were combined (106). This finding was consistent with animal (107, 108) and human neuroimaging data (109–112) suggesting that THC facilitates extinction learning, which is thought to occur during exposure treatment for OCD (113). Thus, THC may have therapeutic benefit to individuals with OCD when paired with exposure treatment.

Based on these findings, in an upcoming fMRI study, we will test the hypothesis that nabilone facilitates extinction learning by impacting relevant brain circuitry. In a separate study, we will also assess whether anxious individuals respond similarly to those with OCD following acute cannabis challenge (i.e., experience smaller anxiety reductions with active cannabis vs. placebo). Using a similar human laboratory design, we will examine the acute effects of smoked cannabis on self-reported anxiety, physiological response to threat, and intoxication in adults with anxiety disorders and high trait anxiety. These novel research directions demonstrate how human laboratory paradigms can guide clinical and translational research involving the effects of cannabis and cannabinoids in psychiatric illness, whether results are positive or negative.

## Discussion

Human laboratory models have been used to understand why individuals use cannabis, to define factors that may contribute to CUD, and to test potential treatments for problematic cannabis use. Applying these procedures can also help elucidate the relationship between cannabis use and psychiatric disorders. Laboratory methods permit controlled administration of cannabis under blinded conditions and assessment of interactions between psychiatric symptoms and discrete cannabis-related outcomes (e.g., intoxication, positive and negative reinforcement, dose-dependency, and tolerance). Finally, the human laboratory can be a powerful translational venue in which to screen potential applications of cannabis or its constituents to treat psychiatric symptoms, evaluate treatments for comorbid psychiatric illness and CUD, and identify cannabis-drug interactions.

A key strength of laboratory models is that they can resolve the acute effects of cannabis on discrete behavioral (e.g., self-administration, choice of non-cannabis rewards), psychological (i.e., self-reported or clinically-assessed symptoms), physiological (e.g., cardiovascular and pharmacokinetic measures), and neurocognitive outcomes (e.g., performance on computerized cognitive tasks, neuroimaging assessment). Laboratory researchers can explore endpoints that are directly related to cannabis use (e.g., models of cannabis relapse) and those that are not (e.g., performance on a social-stress paradigm) (114), and can incorporate both subjective (i.e., self-report) and objective (e.g., physiological) assessments. This ability to test cannabis effects across various levels of analysis is consistent with the US National Institute of Mental Health (NIMH) Research Domain Criteria (RDoC) (115) and other initiatives aimed at developing more objective measurements of psychopathology (116). Moreover, by incorporating fMRI and other neurobiological measures (73), laboratory models might reveal targets to index cannabis effects that could then be explored in future treatment studies. Thus, the goals and designs of human laboratory research are also well-matched to experimental medicine approaches to psychiatric treatment development (117).

Of course, human laboratory research is not without limitations. First, while tight control over various confounding factors is a key strength of laboratory paradigms, this may also limit their generalizability, as real-world settings are rarely so well-regulated. Whether laboratory studies accurately capture cannabis effects on psychopathology or predict medication efficacy also depends on the chosen design and outcome measures. For example, a study of cannabis effects in specific phobia that does not incorporate symptom provocations may fail to detect an anxiolytic signal even when one exists (since patients with specific phobia typically have minimal anxiety in the absence of phobic triggers). In contrast, a finding that cannabis acutely reduces scores on the Depression, Anxiety, and Stress Scale; DASS) in patients with GAD may lead investigators to conclude that cannabis has anxiolytic effects, when in fact participants misinterpreted reduced stress and tension as reflecting anxiety relief (as prior studies in cannabis users suggest they may do) (118).

Second, participant selection is critical to consider given that the risks of cannabis are different for individuals with different psychiatric disorders: Adults with GAD may be at relatively low risk from participating in a study modeling acute effects from smoking one cannabis cigarette, but the same paradigm would involve different risks and ethical concerns in children with GAD (e.g., increased risk for psychosis) or adults with panic disorder (e.g., panic attacks). Even among participants with the same disorder, individual factors like age (92), gender (67, 93), or genetics (94, 95) may influence the response to cannabis and need to be considered when designing studies. Beyond participant selection, volunteers for laboratory studies may differ from general psychiatric populations in important ways: For example, they tend to have fewer medical comorbidities in order to pass inclusion criteria allowing cannabis to be safely administered (98). Moreover, individuals motivated to participate in a cannabis study presumably have neutral or positive expectations about its effects, which could positively bias study results.

Finally, in the US, only cannabis produced by NIDA can be used in human subjects research (14). Yet the available NIDA cannabis varietals differ substantially in their phytocannabinoid contents compared to cannabis available in the community through both legal (119) and illicit means (120). In particular, THC concentrations on average are lower with NIDA cannabis, which has raised concerns about the generalizability of research involving NIDA preparations. However, there are dozens of studies showing that daily, heavy cannabis users (who are presumably tolerant to THC) become intoxicated and show reliable increases in heart rate after smoking NIDA cannabis (41). Thus, despite differences in cannabinoid content between NIDA and community-obtained cannabis, human laboratory models may nonetheless provide clinically-relevant information about cannabis effects in human subjects.

## Conclusion

In summary, human laboratory procedures have a rich history in the field of substance use research. Laboratory methods can also be applied to examine how psychopathology relates to cannabis use, clarify the risks and benefits of cannabis use to individuals with psychiatric disorders, and screen for potential applications of cannabis in psychiatric treatment. Exactly which designs and endpoints best capture specific psychopathologies remains to be determined and should be explored. In addition, while placebo-controlled studies in the human laboratory may provide the necessary groundwork to justify future cannabis trials, further research is needed to verify that promising findings from laboratory models of cannabis treatment are indeed replicable in psychiatric clinical trials. Nonetheless, these laboratory models are powerful tools that can address the increasingly critical need to understand the relationship between cannabis use and psychiatric illness. By improving understanding of cannabis' risks, benefits, and potential treatment applications for patients with psychiatric disorders, laboratory models can enhance the way we conceptualize, diagnose, and treat individuals who suffer from both anxiety and other mental health disorders as well as problematic cannabis use.

## Data Availability Statement

The original contributions presented in the study are included in the article/supplementary material, further inquiries can be directed to the corresponding author/s.

## Author Contributions

RK, MH, and HS contributed to the conceptual framework, literature review, methodological design, and manuscript writing and preparation. All authors contributed to the article and approved the submitted version.

## Conflict of Interest

The authors declare that the research was conducted in the absence of any commercial or financial relationships that could be construed as a potential conflict of interest.
